# Identification of prognostic chromatin-remodeling genes in clear cell renal cell carcinoma

**DOI:** 10.18632/aging.104170

**Published:** 2020-11-20

**Authors:** Yujing Yang, Chengyuan Wang, Ningde Wei, Ting Hong, Zuyu Sun, Jiawen Xiao, Jiaxi Yao, Zhi Li, Tao Liu

**Affiliations:** 1 Department of Medical Oncology, The First Affiliated Hospital of China Medical University, Shenyang 110001, P.R. China; 2 Department of Urology, The First Affiliated Hospital of China Medical University, Shenyang 110001, P.R. China; 3 Department of Medical Oncology, Shenyang Fifth People Hospital, Tiexi District, Shenyang 110001, P.R. China

**Keywords:** clear cell renal cell carcinoma (ccRCC), chromatin-remodeling genes, prognostic biomarkers, BPTF, SIN3A

## Abstract

The aim of this study was to investigate the effects of chromatin-remodeling genes on the prognosis of patients with clear cell renal cell carcinoma (ccRCC). In TCGA-KIRC patients, two subgroups based on 86 chromatin-remodeling genes were established. The random forest algorithm was used for feature selection to identify BPTF, SIN3A and CNOT1 as characterized chromatin remodelers in ccRCC with good prognostic value. YY1 was indicated to be a transcription factor of genes highly related to BPTF, SIN3A and CNOT1. Functional annotations indicated that BPTF, SIN3A, CNOT1 and YY1 are all involved in the ubiquitin-mediated proteolysis process and that high expression of any of the five associated E3 ubiquitin ligases found in the pathway suggests a good prognosis. Protein network analysis indicated that BPTF has a targeted regulatory effect on YY1. Another independent dataset from International Cancer Genome Consortium (ICGC) showed a strong consistency with results in TCGA. In conclusion, we demonstrate that BPTF, SIN3A and CNOT1 are novel prognostic factors that predict good survival in ccRCC. We predicted that the good prognostic value of chromatin-remodeling genes BPTF and SIN3A is related to the regulation of YY1 and that YY1 regulates E3 ubiquitin ligases for further degradation of oncoproteins in ccRCC.

## INTRODUCTION

Clear cell renal cell carcinoma (ccRCC), which accounts for 70-80% of all renal cell carcinoma (RCC) patients, is one of the most lethal malignancies of the urinary system [1]. The genetic changes underlying ccRCC include alterations in genes regulating the hypoxia pathway (for example, VHL) and maintaining chromatin states (for example, PBRM1 and ARID1A) [2]. Prognostic biomarkers play an important role in stratifying patients to avoid both overtreatment and inadequate treatment of ccRCC [3]. Currently, numerous studies on the hypoxia pathway have been reported, but few prognostic factors are widely accepted in ccRCC. Recent studies have shown that ARID1 and PBRM1 have significant prognostic value and distinguish the sensitivity of patients to therapy in RCC [4–9]. Interestingly, both ARID1 and PBRM1 are chromatin-remodeling genes. This strongly suggests that chromatin-remodeling genes are crucial in determining the prognosis and guiding the treatment of ccRCC. Therefore, the exploration of novel prognostic biomarkers involved in chromatin remodeling in ccRCC is urgently required.

Chromatin remodeling is the process of dynamic modification of the chromatin structure to control gene expression by allowing regulatory transcription proteins access to condensed DNA. In addition, chromatin remodeling plays epigenetic regulatory roles in many processes related to cancer development, including cell cycle progression, cell death, cell pluripotency, and DNA repair [10–12]. During DNA replication, DNA repair and transcription, the chromatin structure is continuously modified, thereby exposing specific gene regions and allowing DNA-interacting enzymes access to specific regions of DNA. Chromatin remodelers play critical roles in stem and progenitor cell differentiation, lineage commitment and organogenesis during mammalian development [13, 14]. With advances in gene sequencing and an in-depth understanding of the epigenetic regulation of DNA-templated processes, numerous studies have indicated that extensive dysregulation of chromatin remodelers and the resulting inappropriate expression of regulatory genes together lead to oncogenesis [15–18]. Therapeutic targeting of chromatin remodelers has been shown to be effective in controlling tumorigenesis [12]. Furthermore, the prognostic value and therapeutic decision-making significance of chromatin remodelers in ccRCC have been demonstrated [5, 6, 19, 20]. In recent years, chromatin-remodeling genes, including ARID1, PBRM1, BAP1, SETD2, bromodomain PHD-finger transcription factor (BPTF), and SMARCA4, have been the focus of research in various types of tumors [4, 21–24]. However, the prognostic value of chromatin-remodeling genes as an overall indicator has not been evaluated in many types of tumors. Hence, in this study, we explored a prognostic model based on chromatin-remodeling genes and evaluated the prognostic value of several characterized chromatin-remodeling genes in ccRCC.

In our study, we downloaded data from The Cancer Genome Atlas (TCGA) database, International Cancer Genome Consortium (ICGC) and Human Transcription Factor Database (HumanTFDB). Then, a prognostic model based on chromatin-remodeling genes was established, and characterized chromatin-remodeling genes were identified in ccRCC. In this paper, the prognostic value of chromatin-remodeling genes and the associated mechanism in ccRCC were explored. The study of chromatin-remodeling genes provides new insight into the prognostic biomarkers and mechanisms of ccRCC.

## RESULTS

### Identification of subgroups with distinct prognoses in TCGA-KIRC patients based on consensus clustering

**Figure 1:**
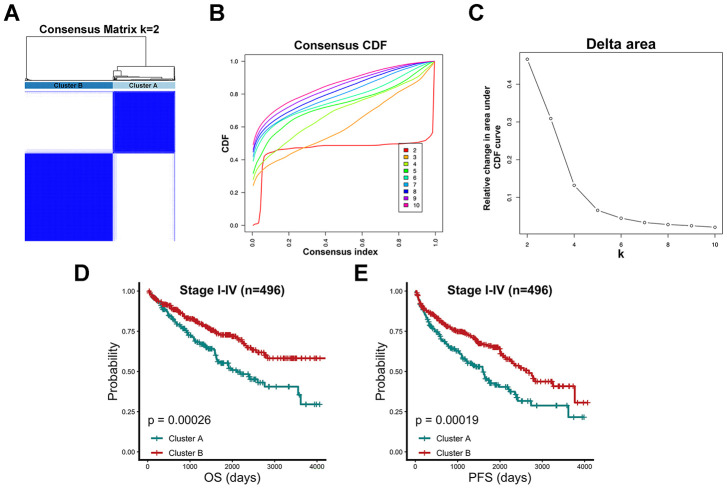
**Consensus clustering algorithm identified two clusters with prognostic value.** (**A**) 496 TCGA-KIRC patients were group into a consensus matrix with Cluster A (n=206) and Cluster B (n=290) based on 86 chromatin-remodeling factors and consensus clustering algorithm. (**B**) Consensus matrix among cumulative distribution function (CDF) curves from 2 to 10 clusters. (**C**) Relative changes in delta area under the CDF curve for each k category from 2 to 10. (**D**) Comparison of overall prognostic differences between Cluster A and Cluster B upon all stage patients. (**E**) Comparison of progression-free prognostic differences between Cluster A and Cluster B upon all stage patients.

After data processing, a total of 496 TCGA-KIRC patients were included, and 86 chromatin-remodeling genes were accepted as the definitive input in a first-step consensus clustering analysis. According to the comparison of the cumulative distribution function (CDF) curve from 2 to 10 category numbers (Figure 1B, 1C), two major clusters (cluster A and cluster B) were identified for which model stabilization became the highest with the optimal classification accuracy of 2 categories in the consensus matrix (Figure 1A). Patients belonging to cluster B (n=290) exhibited significantly better prognostic values for overall survival (OS) (P= 0.00026) and progression-free survival (PFS) (P= 0.00019), while patients in cluster A (n=206) displayed a strong tendency for poor clinical outcomes (Figure 1D, 1E). Univariate and multivariate Cox regression analyses indicated that in addition to age, clinical stage and differentiation grade, cluster B was a significant factor correlated with OS and PFS endpoints in TCGA-KIRC patients (hazard ratio (HR): 0.667; 95% confidence interval (CI): 0.535-0.831; P <0.001; Table 1). As the two clusters show substantial differences in survival outcomes, their associations with clinical parameters and vital mutations were also estimated. Clearly, cluster B was significantly related to a relatively low clinical stage and good histological differentiation (Supplementary Table 1). However, the statuses of some top mutant genes in renal carcinoma patients, including VHL, PBRM1, TP53 and MTOR, which were collected from the Catalogue of Somatic Mutations in Cancer (COSMIC) database, did not show evident differences between the two clusters.

**Table 1:** Univariate and multivariate cox regression of Cluster A and Cluster B for overall survival and progression-free survival in TCGA-KIRC (n=496).

**Variable**		**Univariate analysis**		**Multivariate analysis**	
		**HR (95% CI)**	**P value**	**HR (95% CI)**	**P value**
**TCGA-KIRC OS (N=496)**					
**Cluster**	Cluster B vs Cluster A	0.667(0.535-0.831)	< 0.001	0.767(0.613-0.96)	0.02
**Age**	Continuous	1.03(1.017-1.044)	< 0.001	1.032(1.018-1.047)	< 0.001
**Gender**	Male vs Female	0.961(0.766-1.206)	0.731		
**Stage**	Stage IV vs Stage I-III	3.037(2.419-3.812)	< 0.001	2.672(2.102-3.398)	< 0.001
**Grade**	Grade III-IV vs Grade I-II	2.06(1.595-2.661)	< 0.001	1.565(1.196-2.048)	0.001
**TCGA-KIRC PFS (n=496)**					
**Cluster**	Cluster B vs Cluster A	0.695(0.573-0.843)	< 0.001	0.799(0.656-0.973)	0.025
**Age**	Continuous	1.021(1.009-1.032)	< 0.001	1.021(1.009-1.033)	< 0.001
**Gender**	Male vs Female	1.118(0.912-1.371)	0.282		
**Stage**	Stage IV vs Stage I-III	3.788(3.073-4.668)	< 0.001	3.431(2.761-4.263)	< 0.001
**Grade**	Grade III-IV vs Grade I-II	2.011(1.618-2.499)	< 0.001	1.652(1.32-2.069)	< 0.001

### Characterized chromatin-remodeling factor screening by the random forest algorithm

**Figure 2:**
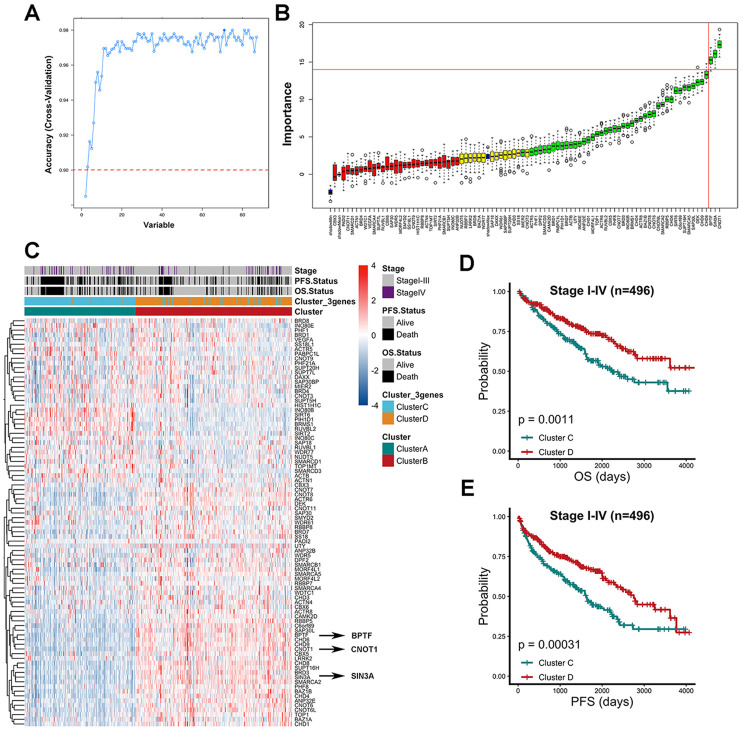
**Characterized chromatin-remodeling factors selection based on random forest algorithm.** (**A**) The relationship between the number of variables and model accuracy after multiple cross-validations of the random forest process was shown. The auxiliary line was added at 0.9 accuracy and the first point indicated 2 variables while the following points added one variable compared to the previous one. (**B**) The importance of each variable in model contribution was shown. The horizontal axis represents the position of 86 chromatin-remodeling factors in the rank of high to low importance from right to left. Red features were confirmed unimportant, yellow features were designated as tentative and green features were confirmed important. Three blue boxplots mapped to minimal, mean and maximum Z scores of shadow attributes respectively. (**C**) Z score gene expression level of 86 chromatin-remodeling factors among consensus cluster A and cluster B. Clinicopathological stage, outcome status and groups (cluster C and cluster D) of consensus cluster based on BPTF, CNOT1, and SIN3A were annotated in the heatmap. (**D**) Comparison of overall prognostic differences between Cluster C and Cluster D upon all stage patients. (**E**) Comparison of progression-free prognostic differences between Cluster C and Cluster D upon all stage patients.

After multiple cross-validations of the random forest (RF) process, the relationship between the number of selected variables and model accuracy based on the consensus cluster A and cluster B grouping was evaluated (Figure 2A). The cut-off value of the model’s accuracy was defined as 0.9, and the accuracy reached 90% when at least 3 variables were included in the model (Supplementary Table 2). The model achieved its highest accuracy when 38 variables were included by rank, and the top three variables were CNOT1, SIN3A and BPTF (Supplementary Table 3), which means that these three factors could represent the original chromatin-remodeling model with 90% accuracy. According to the RF algorithm, the importance of the contributions of all 86 chromatin-remodeling factors in the model was also calculated (Figure 2B). The top three variables of the green features, which are features designated as having robust importance, were CNOT1, SIN3A and BPTF. Agreement between the two approaches validated the consistency and enhanced the confidence of the result. The Z-score-processed expression data of the 86 chromatin-remodeling factor dendrogram clustering heatmap based on prespecified groups were utilized to discover the differences in CNOT1, SIN3A and BPTF expression levels between cluster A and cluster B (Figure 2C). Consensus clustering based on BPTF, SIN3A and CNOT1 was also performed to obtain cluster C and cluster D. Interestingly, we found that BPTF, SIN3A and CNOT1 were all highly expressed in cluster B, a group previously associated with relatively good outcomes, and the patient proportions and distributions of cluster C and cluster D were consistent with those of cluster A and cluster B. Then, comparisons of OS and PFS between cluster C and cluster D for the 496 TCGA-KIRC patients were performed to verify conformity. As shown in Figure 2D, 2E, compared with those in cluster C, the ccRCC patients in cluster D had better OS (P= 0.0011) and PFS (P= 0.00031). The risk-predicted accuracy of the combined model including clinical stage, BPTF, SIN3A and CNOT1 was higher than that of the univariate clinical stage model for one-year, three-year and five-year OS (Supplementary Figure 1A, 1B). The prediction of long-term survival varied even more between the two models. In summary, the discovery that a model based on BPTF, SIN3A and CNOT1 could faithfully represent the model resulting from the original input of chromatin-remodeling factors was confirmed in a variety of ways.

### Survival analysis and correlation analyses of clinical parameters with BPTF, SIN3A and CNOT1

**Figure 3:**
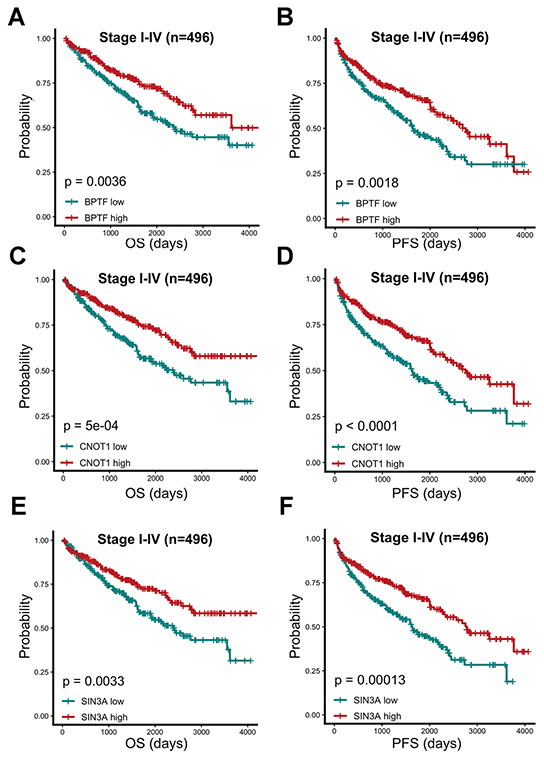
**Prognostic analysis of three characterized chromatin-remodeling factors upon 496 TCGA-KIRC patients.** Comparison of overall and progression-free prognostic differences between two groups divided by BPTF (**A**, **B**), CNOT1 (**C**, **D**) and SIN3A (**E**, **F**) expression levels based on median expression level.

**Figure 4:**
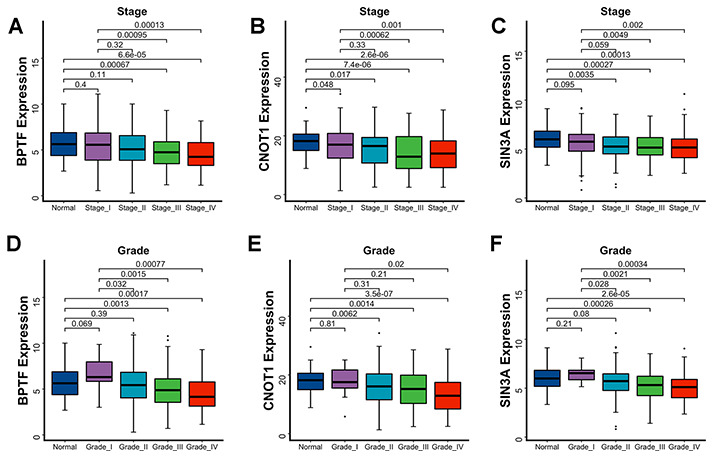
**Association between three characterized chromatin-remodeling factors expression levels and clinicopathological parameters.** Differentiated expression of BPTF, CNOT1 and SIN3A between normal renal tissue, disparate clinicopathological stages (**A**–**C**) and clinicopathological grade (**D**–**F**). The Wilcoxon test was used for group comparison.

The OS and PFS estimations of BPTF, SIN3A and CNOT1 were performed for 496 TCGA-KIRC patients. After dividing ccRCC patients into three equal quantile divisions based on selected gene expression, we found that compared with the low expression group, the high BPTF expression group exhibited better OS (P< 0.0001) and PFS (P< 0.0001) (Figure 3A, 3B). Likewise, ccRCC patients with high CNOT1 expression had better OS (P< 0.0001) and PFS (P= 0.00018) than those with low expression (Figure 3C, 3D), and patients in the high SIN3A expression group also had better OS (P= 0.00081) and PFS (P= 0.00015) outcomes than those in the low expression group (Figure 3E, 3F). Associations between the expression levels of these three characterized chromatin-remodeling genes and clinicopathological parameters were also evaluated. As the clinical stage advanced, the expression levels of BPTF, SIN3A and CNOT1 appeared to decrease gradually (Figure 4A–4C). In regard to differentiation grade, significantly reduced expression was found in poorly differentiated tumor tissue compared with normal renal tissue or neoplastic tumor tissue with a level I grade (Figure 4D–4F).

### Functional annotation of CNOT1, SIN3A and BPTF and property analysis of their highly correlated genes

**Figure 5:**
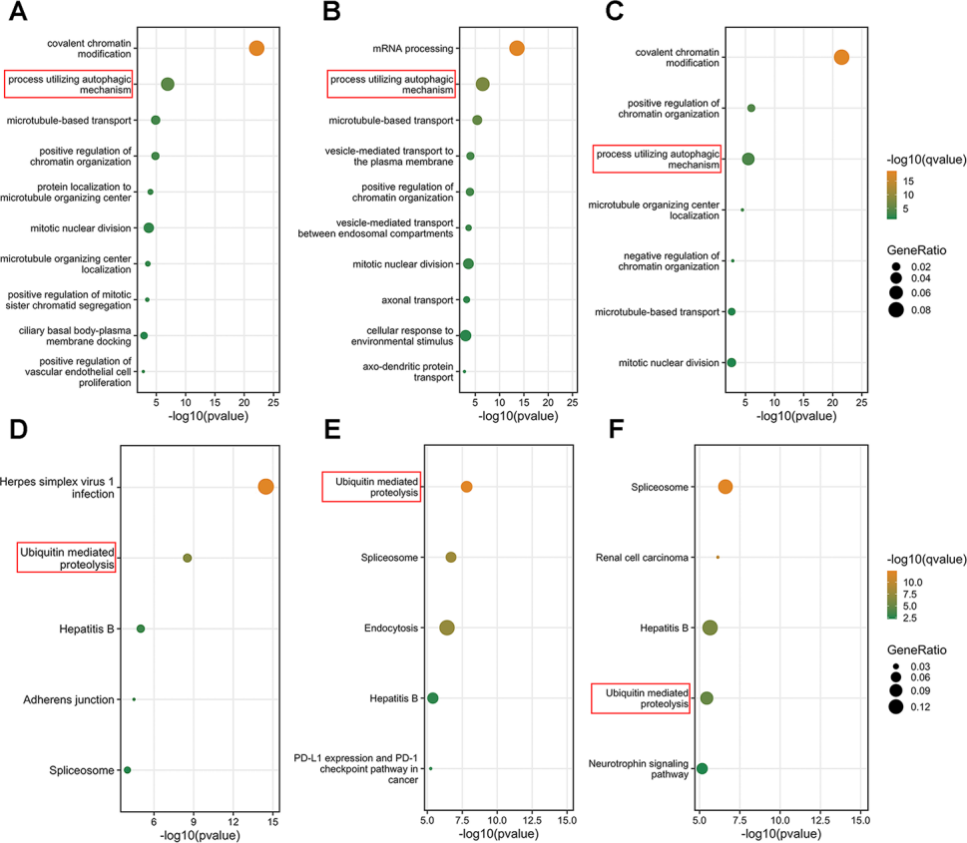
**Function prediction of three characterized chromatin-remodeling factors.** (**A**–**C**) Top 10 biological process enrichment results of genes in which correlation with BPTF, CNOT1 or SIN3A were greater than 0.5. (**D**–**F**) Top 5 KEGG pathway enrichment results of genes which correlation with BPTF, CNOT1 and SIN3A were greater than 0.5. The results were ranked by the enrichment scores and displayed corresponding to -log10(q-value) and gene ratio enriched in the selected pathways.

**Figure 6:**
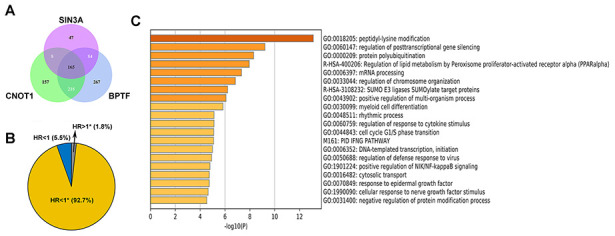
**The properties of the intersected genes.** (**A**) Venn diagram shows the number of intersection genes of genes in which correlation with BPTF, CNOT1, and SIN3A was greater than 0.6. (**B**) Univariate Cox regression results for the overall survival of all 165 intersected genes were displayed by ratio pie plot. "*" means the statistic P-value was significant (P<0.05). (**C**) Top 20 results of gene ontology analysis upon all 165 intersected genes.

Since the results of the survival analysis and correlation analysis between clinicopathological parameters and BPTF, SIN3A and CNOT1 in TCGA-KIRC were highly consistent, we investigated the potential mechanisms of these three chromatin-remodeling genes and their highly correlated genes (Pearson r>0.5; P<0.001) in ccRCC. As shown in Figure 5A–5C, gene ontology (GO) analysis indicated that BPTF, SIN3A and CNOT1 function in the biological process of chromatin regulation, which is consistent with current knowledge. In addition, some of their correlated genes were found to be involved in the process of utilizing the autophagic mechanism. Furthermore, the top 5 Kyoto Encyclopedia of Genes and Genomes (KEGG) pathway enrichment analysis terms indicated that genes associated with ubiquitin-mediated proteolysis pathways were relatively enriched (Figure 5D–5F). Because the genetic annotations and gene sets both largely associated with protein degradation pathways, we focused on the properties of their intersected genes. The Venn diagram shown in Figure 6A illustrates the number and overlap of the significantly highly correlated genes of BPTF, SIN3A and CNOT1 (Pearson r>0.6; P<0.001) in ccRCC patients. Univariate Cox regression analysis of OS was utilized to evaluate the prognostic value of 165 intersected genes. We found that the majority of these genes (92.7%; 153/165) significantly predicted a good prognosis, while only a small group of them (1.8%; 3/165) were significantly associated with poor survival outcomes or were nonsignificant (5.5%; 9/165) in ccRCC (Figure 6B). Comprehensive GO analysis performed on the 165 intersected genes suggested that processes related to posttranscriptional gene silencing regulation and protein polyubiquitination were the top enriched processes (Figure 6C). An interactive network of the top 15 enriched pathways among the intersected genes determined by KEGG analysis was constructed, and it was very consistently found that ubiquitin-mediated proteolysis pathways ranked as the second highest score in the enrichment analysis (Supplementary Figure 2A). Hence, we hypothesized that BPTF, SIN3A and CNOT1 all play roles in the occurrence and development of ccRCC by affecting autophagy regulation and activating ubiquitin-mediated proteolysis, which might account for their good prognostic value.

### Prediction of YY1 as a transcriptional factor of the intersected genes

**Figure 7:**
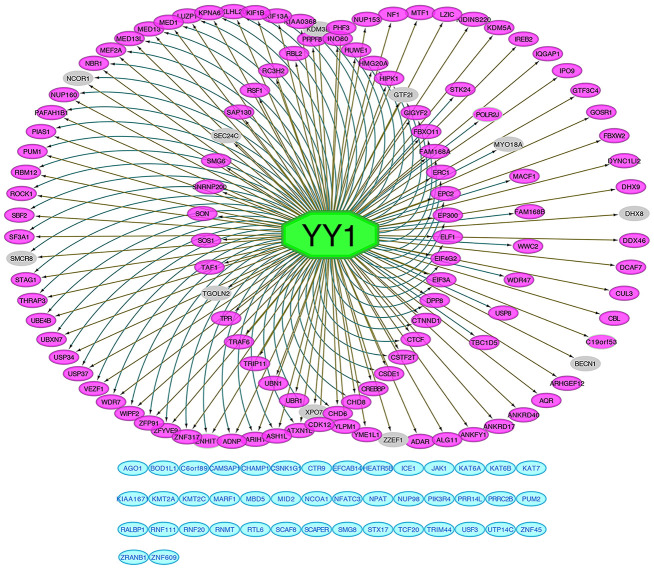
**Prediction of YY1 as intersected genes regulator.** All 165 intersected genes predictive network of transcription factor YY1 performed by iRegulon. Genes in the left circle were found regulated by YY1 according to both motif's matching and ChIP-seq tracks signals. Genes in the right circle were found regulated by YY1 based on ChIP-seq tracks signals. Genes in the inner right circle were found regulated by YY1 based on motif's matching. Genes in blue modules were not mapped to YY1. Genes in grey color are those that are less than 0.3 correlated with YY1.

The iRegulon plugin was used to predict the putative transcription factors of all 165 intersected genes with Cytoscape Java software. According to iRegulon, YY1 was the most likely transcription factor of these genes, and the predictive results of other regulators were not sufficient with strong evidence. After excluding genes that did not match YY1 (44/165; blue module) and genes with a correlation factor less than 0.3 with YY1 by Pearson’s correlation analysis (11/165; gray module), the remaining genes showed at least two evidence-based relationships with YY1, including enriched motif mapping information obtained from the Encyclopedia of DNA Elements (ENCODE), JASPAR and HOMER databases, ChIP-seq track signal calculations and correlation analysis (Figure 7). Four out of the top 10 enriched pathways identified by KEGG analysis were verified by single-gene YY1 gene set enrichment analysis (GSEA), the TGF-beta signaling pathway, the Wnt signaling pathway, RCC and ubiquitin-mediated proteolysis (Supplementary Figure 2B); these results were consistent with the enrichment results for the intersected genes (Supplementary Figure 2A), which could be considered additional evidence supporting YY1 as the potential transcriptional factor due to the similar pathway annotations. Five E3 ubiquitin ligase genes, CBL, UBE4B, TRAF6, HUWE1, and PIAS1, that were enriched in the ubiquitin-mediated proteolysis pathway identified by the previous interactive KEGG analysis (Supplementary Figure 2A), were predicted to be regulated by YY1. Because ubiquitin E3 ligases have an impact on almost every aspect of eukaryotic biological processes by promoting protein ubiquitination and degradation, it was necessary to predict the possible substrates of CBL, UBE4B, TRAF6, HUWE1 and PIAS1 to elucidate their mechanisms. Based on five relationship predictive methods provided by the UbiBrowser online tool, some potential targets were found and are listed in Supplementary Figure 3, and many of them are best known as oncoproteins in multiple cancer types, such as proteins in the JAK family, AKT1 and BRAF. As shown in Supplementary Figure 4, OS and PFS analyses with patients stratified by the median cut-off values for the five E3 ubiquitin ligases were also performed for the 496 TCGA-KIRC patients. Obviously, patients with high CBL, UBE4B, TRAF6, HUWE1 or PIAS1 expression exhibited better ccRCC outcomes than those with corresponding low expression.

### Relationship predictions for YY1 and the characterized chromatin remodelers

**Figure 8:**
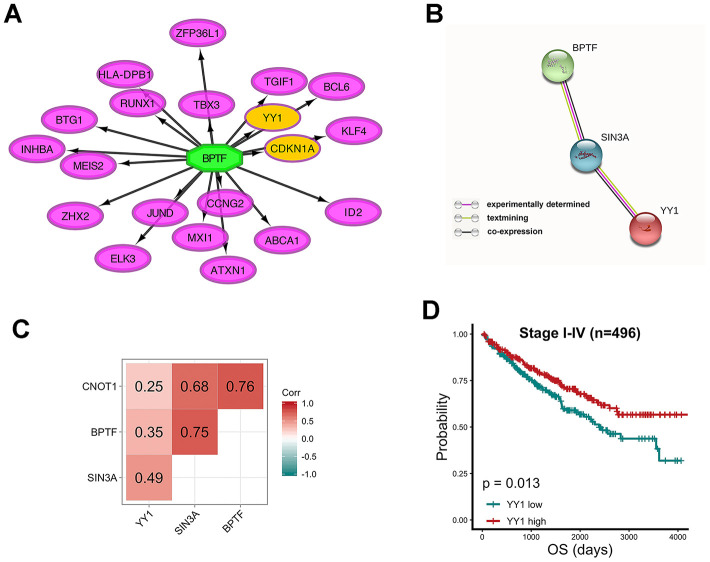
****Relationship between characterized chromatin-remodelers and YY1 (**A**) Top 20 predicted target genes of BPTF. (**B**) The interactive relationship between BPTF, SIN3A and YY1 produced by STRING. (**C**) Pearson correlation between the expression levels of BPTF, CNOT1, SIN3A and YY1 upon 496 TCGA-KIRC patients. (**D**) Comparison of overall prognostic differences between YY1 high and low expression groups by median cut-off value.

Another function encoded in iRegulon was exploited to query the targets of BPTF, a transcription-associated chromatin remodeler. According to MSigDB, GeneSigDB and Ganesh Clusters, YY1 and CDKN1A were predicted to be in the top 20 matching results (Figure 8A). Studies have identified CNKN1A as a target gene of YY1 that binds to the BRCA2 protein to have a role in tumor suppression [25]. Through experimental determination and text mining performed with the STRING database, confirmed co-expression relationships were found among the expression of BPTF, SIN3A and YY1 with SIN3A acting as the crosslinking bridge, but CNOT1 did not show any connectivity in the protein network (Figure 8B). One-year, three-year and five-year receiver operating characteristic (ROC) curves of a combined model including clinical stage, BPTF, SIN3A and CNOT1 and YY1 were constructed, and by comparison, this model had better risk-prediction performance than the previous two models, especially for long-term OS (Supplementary Figure 1A, 1B). The results of Pearson’s correlation analyses of BPTF, SIN3A, CNOT1 and YY1 in TCGA-KIRC patients were all positive and significant, and only the coefficient between CNOT1 and YY1 was relatively low (Figure 8C), which was consistent with the protein interaction results. Significantly positive correlations among BPTF, SIN3A, CNOT1 and YY1 could also be acquired with a TCGA pan-cancer analysis performed by Gene Expression Profiling Interactive Analysis (GEPIA) (Supplementary Figure 5); however, their prognostic values, which were consistent in ccRCC, were not as consistent across other cancer types (Supplementary Table 4). Finally, high expression of YY1 was associated with relatively good OS in TCGA-KIRC patients (Figure 8E), which implied that there might be a distinct interaction relationship among BPTF, SIN3A, CNOT1 and YY1 in ccRCC patients that leads to an unclear tumor-suppressive function that promotes improved clinical outcomes.

### Validation in ICGC dataset

We downloaded another RNA-Seq dataset from ICGC which containing 91 patients with renal cancer for validation. Patients in ICGC dataset can be divided into 3 groups with differential expression of BPTF, CNOT1 and SIN3A by consensus clustering. Patients with low BPTF, CNOTA and SIN3A expression have significantly worse OS and no pathways were enriched from KEGG database. Patients with relatively median or high expression of BPTF, CNOT1 and SIN3A suffer better OS and ubiquitin-mediated proteolysis pathway was enriched (NES=1.78, P=0.000) as one of the top 10 enrichment, which was consistent with the result acquired from TCGA (Supplementary Figure 6). Furthermore, patients with high expression level of BPTF and SIN3A have significantly better OS but the result of CNOT1 was insignificant. The expression levels of BPTF, CNOT1 and SIN3A significantly decreased in stage IV patients compared with stage I patients. Since BPTF, CNOT1 and SIN3A were identified as protective factors in the progression of ccRCC in TCGA dataset, that conclusion was validated in ICGC dataset (Supplementary Figure 7). Using iRegulon plugin of cytoscape, YY1 was identified as the most likely predicted transcriptional factor of overlapped correlated genes according to both motif's matching and ChIP-seq tracks signals. Pearson correlation between the expression of BPTF, CNOT1, SIN3A, YY1 and the 5 E3-ligases were significantly high, which suggested closed relationships of them. High expression of CBL and HUWE1 indicated significantly better OS while the similar prognostic tendency could be obtained in PIAS1, TRAF6 and UBE4B but were not significant. The results of transcriptional factor's prediction, correlation analysis and survival analysis in ICGC dataset were consistent with TCGA (Supplementary Figure 8).

## DISCUSSION

In this study, based on 86 chromatin-remodeling genes, two subgroups of TCGA-KIRC patients with distinct prognoses were defined by consensus clustering of ccRCC samples. The clustering of three genes (BPTF, SIN3A and CNOT1) could effectively represent 90% of chromatin-remodeling genes and indicate the prognosis of ccRCC. Furthermore, each of the three genes was also identified to be indicative of a good prognosis, and high expression was related to relatively low clinical stage and good histological differentiation in ccRCC. The results of a functional analysis indicated that BPTF, SIN3A and CNOT1 were all involved in ubiquitin-mediated proteolysis in ccRCC. In addition, YY1 was indicated to be an important transcription factor of genes highly related to BPTF, SIN3A and CNOT1. Correlation and protein network analyses indicated that BPTF, SIN3A and CNOT1 were highly correlated with YY1 and that YY1 might be a regulatory target of BPTF and co-express with SIN3A in ccRCC. Furthermore, another RNA-Seq dataset from ICGC which containing 91 patients with renal cancer was used to validate the results, indicating the results in TCGA were not database-specific. The highlight of our study is the first demonstration that BPTF, SIN3A, CNOT1 and YY1 are novel biomarkers of good prognosis in ccRCC.

BPTF and SIN3A are new prognostic biomarkers for predicting survival in ccRCC. BPTF is the largest subunit of the nucleosome-remodeling factor (NURF) complex and plays a vital role in chromatin remodeling for gene activation through its association with histone acetylation or methylation [20, 26]. The important roles of BPTF in thymocyte maturation, embryonic development, and T cell homeostasis and function have been confirmed [27–30]. Recent studies have indicated that BPTF promotes tumor cell growth in several types of cancers [26, 31–35]. Interestingly, in this paper, BPTF was first identified as a gene positively associated with a good prognosis in ccRCC. SIN3A is a core component of the histone deacetylation activity-associated transcriptional repressor complex [36]. In mammals, Sin3 proteins can recruit histone deacetylases (HDACs) to chromatin-bound transcription factors to repress the expression of target genes [37]. SIN3A regulates processes important for development and homeostasis including mitochondrial biogenesis, cell death and neuronal fate selection [38, 39]. In breast cancer cells, interference with SIN3A function induces epigenetic reprogramming and differentiation, and the SIN3A/HDAC complex plays an important role in maintaining sensitivity to chemotherapy in breast cancer [40, 41]. In this study, we demonstrated for the first time that high SIN3A expression suggests a good prognosis in ccRCC. Consistent with high BPTF or SIN3A expression suggesting a good prognosis in ccRCC, we confirmed that high expression of BPTF or SIN3A was significantly associated with a relatively low clinical stage and good histological differentiation in ccRCC, respectively. According to the TCGA database and existing studies, BPTF and SIN3A in most types of tumors have no prognostic value. Thus, the clear mechanistic understanding of the unique prognostic significance of BPTF and SIN3A in ccRCC needs to be further developed.

In our study, BPTF, SIN3A and CNOT1 were all involved in processes utilizing the autophagic mechanism and ubiquitin-mediated proteolysis in ccRCC. However, how these three genes participate in the process of ubiquitination remains unknown. Ubiquitination is an important posttranslational modification that controls many biological processes, such as cell division and differentiation, in all eukaryotes, and it also controls many steps in autophagy [42]. Furthermore, autophagy deficiency induces inhibition of histone ubiquitination [43]. These results are consistent with high expression of BPTF, SIN3A and CNOT1 indicating a good prognosis in ccRCC. We predict that BPTF, SIN3A and CNOT1 enhance genomic stability and inhibit tumor cell formation by positively regulating ubiquitination in ccRCC.

YY1 was identified as the intersected gene regulator in the network based on chromatin-remodeling genes highly related to BPTF, SIN3A and CNOT1. In ccRCC, YY1 was identified as a novel biomarker of a good prognosis.

YY1, a ubiquitously expressed transcription factor, plays an essential role in early embryogenesis, adult tissue formation and the regulation of Th2 cell and B cell differentiation [44–47]. However, the mechanism underlying YY1 involvement in ccRCC progression has not been reported. Interestingly, like BPTF, SIN3A and CNOT1, YY1 is also enriched in ubiquitin-mediated proteolysis. Since YY1 is highly correlated with BPTF, SIN3A and CNOT1 in ccRCC, we conclude that YY1, along with the three chromatin-remodeling genes, is involved in the regulation of ubiquitination.

The possible regulatory effects of BPTF and SIN3A on YY1 were investigated via functional and correlation analyses in our study. Five ubiquitin E3 ligases, CBL, UBE4B, TRAF6, HUWE1 and PIAS1, were predicted to be regulated by YY1. Based on five relationship predictive methods provided by the UbiBrowser online tool, some potential targets were found and many of the substrates of these five E3 ubiquitin ligases, such as proteins in the JAK family, AKT1 and BRAF, are well-known oncoproteins in multiple cancer types. In patients with ccRCC, many of these substrates have been identified as poor prognostic factors, such as: JAK2 [48], JAK3 [49], CSK [50], RET [51], AR [52], AHR [53, 54], IRF4 [55], SHC1 [56], TGFBR2 [57]. Interestingly, consistent with the prognostic value of BPTF, SIN3A, CNOT1, and YY1, high expression of any of the five E3 ubiquitin ligases indicated a good prognosis in ccRCC. In addition, BPTF, SIN3A, CNOT1 and YY1 were highly positively correlated in both pan-cancer and ccRCC datasets. We further mined ccRCC datasets in GEO to verify our conclusions in ccRCC. In GSE36895 containing 29 samples from ccRCC patients, positive correlation was found among BPTF/CNOT1/SIN3A/YY1, in which the correlation between SIN3A and CNOT1/YY1 was significant (data not shown) [58]. This result supports the relationship of BPTF/CNOT1/SIN3A/YY1 found in TCGA. Protein network analysis indicated that BPTF might have a targeted regulatory effect on YY1. Confirmed co-expression relationships were found among BPTF, SIN3A and YY1, with SIN3A acting as the crosslinking bridge. Based on the above results, we predict that BPTF, SIN3A and YY1 are involved in one pathway that is likely associated with ubiquitination in ccRCC. Therefore, we conclude that the good prognostic value of BPTF and SIN3A is caused by their involvement in the regulation of YY1, which further degrades oncoproteins through five E3 ubiquitin ligases in ccRCC.

The Ccr4-Not complex is a highly conserved regulator of mRNA metabolism, and CNOT1 is the large human subunit of the Ccr4-Not complex [59]. CNOT1, a transcriptional repressor, is crucial for maintaining embryonic stem cells in a pluripotent state, and a specific CNOT1 mutation can lead to holoprosencephaly and the novel syndrome of pancreatic agenesis [60]. CNOT1 can target heterochromatin to regulate gene expression and protect genome integrity [61]. In our study, we found that CNOT1 played an important role in chromatin remodeling, and we demonstrated for the first time that high CNOT1 expression suggested a good prognosis in ccRCC. Interestingly, like BPTF, SIN3A and YY1, CNOT1 is also involved in ubiquitin-mediated proteolysis. However, the mechanism underlying CNOT1 involvement in ccRCC needs further study, and we hope that more research will focus on CNOT1 in ccRCC.

To evaluate the reproducibility of the results in TCGA, another additional independent RNA-Seq dataset from ICGC which containing 91 patients with renal cancer was used to compare the results to our preceding findings in TCGA dataset. Significantly, a strong consistency between TCGA and ICGC database was found, suggesting the results in our study were solid and not database-specific.

In conclusion, we identified three chromatin-remodeling genes, BPTF, SIN3A and CNOT1, that could predict a good prognosis, which was related to a relatively low clinical stage and good histological differentiation in ccRCC. We confirmed that BPTF, SIN3A, CNOT1 and YY1 are all involved in ubiquitin-mediated proteolysis. We predicted that BPTF and SIN3A may be involved in regulating YY1, which regulates E3 ubiquitin ligases to promote further degradation of oncoproteins in ccRCC. The limitation of this study is the lack of experimental validation. Further experiments are necessary to validate the prognostic value of BPTF, SIN3A, CNOT1 and YY1 in ccRCC.

## MATERIALS AND METHODS

### Datasets preparation

Raw RNA-seq count data, fragment per kilobase per million (FPKM) values and the corresponding clinicopathological features of 611 ccRCC patients were retrieved from the TCGA using TCGAbiolinks R packages version 2.13.6 [62]. After preprocessing to integrate the OS and PFS outcome information obtained from the TCGA clinical data resource [63], patients who met the following criteria were excluded from this study: (1) prior receipt of neoadjuvant therapy; (2) an unclear histopathological definition; (3) missing survival records; and (4) a follow-up period less than 30 days. Thus, a total of 496 tumor cases and 72 normal renal samples satisfying the inclusion criteria were evaluated in the final research. The mutational annotation format (MAF) of somatic mutation data was also downloaded from the TCGA database. A dataset included (reads per kilobase per million) RPKM expression profile of 91 patients with renal cancer and the corresponding clinical information was downloaded from ICGC database (https://icgc.org/) for validation.

### Clustering of chromatin-remodeling factors

We required a list of all 136 chromatin-remodeling genes from the Human Transcription Factor Database (HumanTFDB; http://bioinfo.life.hust.edu.cn/HumanTFDB#!/) after excluding 6 factors in the chromatin Y family to avoid a sex bias and 4 factors in the histone cluster 1 H1, which have relatively low expression levels in renal tumor samples. The paired Wilcoxon test (P < 0.01) was applied to compare differential gene expression between renal cancer tissue and nontumoral tissue samples; subsequently, 105 genes were selected as significant factors related to tumorigenesis. We then took the intersection of these genes and the top 50% variant genes were determined by the median absolute deviation (MAD) of FPKM expression data. Finally, 86 chromatin-remodeling genes were accepted as the definitive input for downstream clustering analysis. Based on the K-Means method and Euclidean distance estimation of the gene expression matrix, a consensus clustering algorithm embedded in the R package ConsensusClusterPlus [64] was performed with the following parameters: the clustering number was no more than 10, the proportion of items and features of clusters were no more than 80%, and the number of subsamples was set as 1000 to define the major differentiated groups between samples. The optimal clustering number was identified according to the CDF curve. The same consensus clustering process was performed in TCGA and ICGC datasets after three characterized chromatin remodeling-associated genes were selected.

### Characterized gene selection

Machining learning RF wrapper approaches implemented in the R packages caret [65] and Boruta [66] were applied as feature selection classifiers. Based on the consensus clustering group, we employed a random resampling iteration procedure performed 10 times with 100-fold cross-validations through the caret package on chromatin-remodeling gene expression data to confirm the accuracy of the prediction and to reduce the variability in model performance. By comparing the importance of the original features with randomly accessible shadow-attributed importance through the Boruta package, nonsignificant or low-contribution features were gradually rejected after 30 rounds of tracing regression, while strong or tentatively confirmed features were sustained to stabilize the result of the selection test. After establishing an integrated risk model of clinical stage and selected chromatin-remodeling genes by multivariate Cox regression, we assessed the one- to five-year predictive accuracy of the computed OS prognostic scores by generating ROC curves and comparing their time-dependent areas under the curve (AUCs). The ROC curves were generated with the “timeROC” R package [67].

### Survival analysis

OS and PFS were defined as the clinical endpoints to evaluate survival outcomes. For survival analysis (Kaplan-Meier) performed with the survival (https://CRAN.R-project.org/package=survival) R package, patients in TCGA and ICGC cohorts were divided into two groups of equal size based on the median expression value of BPTF, CNOT1, SIN3A, YY1, CBL, UBE4B, TRAF6, HUWE1 and PIAS1. The differences between groups were estimated by the log-rank test. Univariate Cox proportional hazard regression was used to test the HR and significance of the prognostic value of the intersected genes. Pan-cancer survival analysis results of TCGA datasets were retrieved from the GEPIA website (http://gepia.cancer-pku.cn).

### GSEA

To mine possible biological pathways in which the selected chromatin-remodeling genes might be enriched, GSEA was performed. We estimated the correlations between these genes and all other genes by scoring the Pearson correlation coefficient. The genes that presented a relatively high correlation over 0.5 with BPTF, SIN3A and CNOT1 were collected as GO: BP and KEGG input gene sets for subsequent enrichment analysis with the clusterProfiler R package [68]. Java desktop GSEA software (version 4.0.1) was used to evaluate the c2.cp.kegg.v6.1.symbols enriched annotation on YY1 expression by ranking the Pearson’s correlation coefficients to produce the gene list and to evaluate the enriched pathways of patients in ICGC dataset with differential expression of BPTF, CNOT1 and SIN3A. To explore the connection between each enriched module, an interactive web-based analytic platform that could construct a visual relational network, NetworkAnalyst (https://www.networkanalyst.ca/NetworkAnalyst/home.xhtml), was utilized to visualize the KEGG pathway results for the intersected genes. GO analysis of the intersected genes was carried out with the Metascape online tool (http://metascape.org).

### Regulator prediction

With Cytoscape Java version 3.5.0 (https://cytoscape.org) software, the iRegulon [69] plugin was used to predict the putative transcription factors of target genes and to generate a regulator interactive network. The signature query parameters were determined by default; the enrichment score threshold was 3, and the maximum false discovery rate (FDR) for motif similarity was strictly defined as 0.001. According to both enriched motifs matching from several databases (including JASPAR, HOMER and Elemento) and ENCODE raw ChIP-seq track signals, some significant predictive transcription factors and their calculated targets were filtered to remain and be ranked on the results panel. Genes in which Pearson’s correlation with the predicted regulators was smaller than 0.3 were shadowed in the network analysis of TCGA dataset. Based on the MSigDB, GeneSigDB and Ganesh Clusters gene set sources, the target genes of selected transcriptional regulators were queried and then visualized in a network form with the Query TF-target database function.

### Protein network construction

The STRING (https://string-db.org) database was used to analyze the coexpression relationships between chromatin remodelers and transcription factors in this study. UbiBrowser (http://ubibrowser.ncpsb.org/ubibrowser/) is a website application that provides a confident prediction of human ubiquitin ligase (E3) and corresponding substrate interactions based on a naïve Bayesian computational framework. The biological evidence that supports the predicted result is derived and combined from multiple approaches: published data, protein orthologs, protein domains, protein motifs and network topology.

### Statistical analysis

The chi-square test and corrected chi-square test (values lower than 5 but larger than 1 prompted calibration for continuity) were applied to intergroup comparisons and to assess associations with clinical features. Pearson’s correlation analysis was used to calculate the correlative relation between selected genes. Univariate and multivariate Cox regression models were created to detect the significant risk and protective factors for patients. The two-sided Wilcoxon rank sum test was performed to measure the differences between two variables. All statistical analyses were conducted with R version 3.6.2, and P values below 0.05 were considered statistically significant.

## Supplementary Material

Supplementary Figures

Supplementary Tables
