# Supplementary material for: Identification of prognostic chromatin-remodeling genes in clear cell renal cell carcinoma
Source: Aging (Albany NY). 2020 Nov 20;12(24):25614–42. doi: 10.18632/aging.104170 (PMC7803503; doi:10.18632/aging.104170)
Supplement: Supplementary Figures [file aging-12-104170-s001.pdf]

SUPPLEMENTARY FIGURES

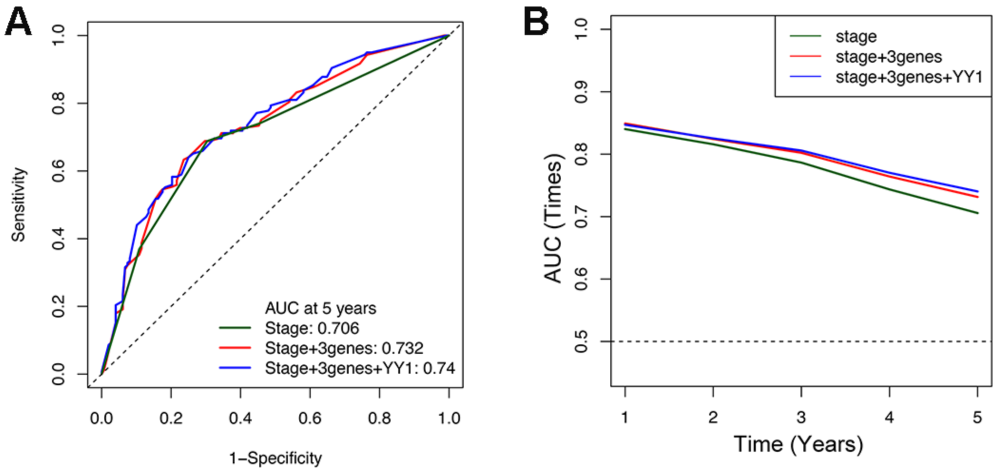

Supplementary Figure 1. Predicted overall survival from one to five years of different models. (**A**) AUC performance of five years survival among clinical stage model, clinical stage+BPTF+CNOT1+SIN3A model and clinical stage+ BPTF+CNOT1+SIN3A+YY1 model. (**B**) Comparison of time-dependent ROC curves of three different models.

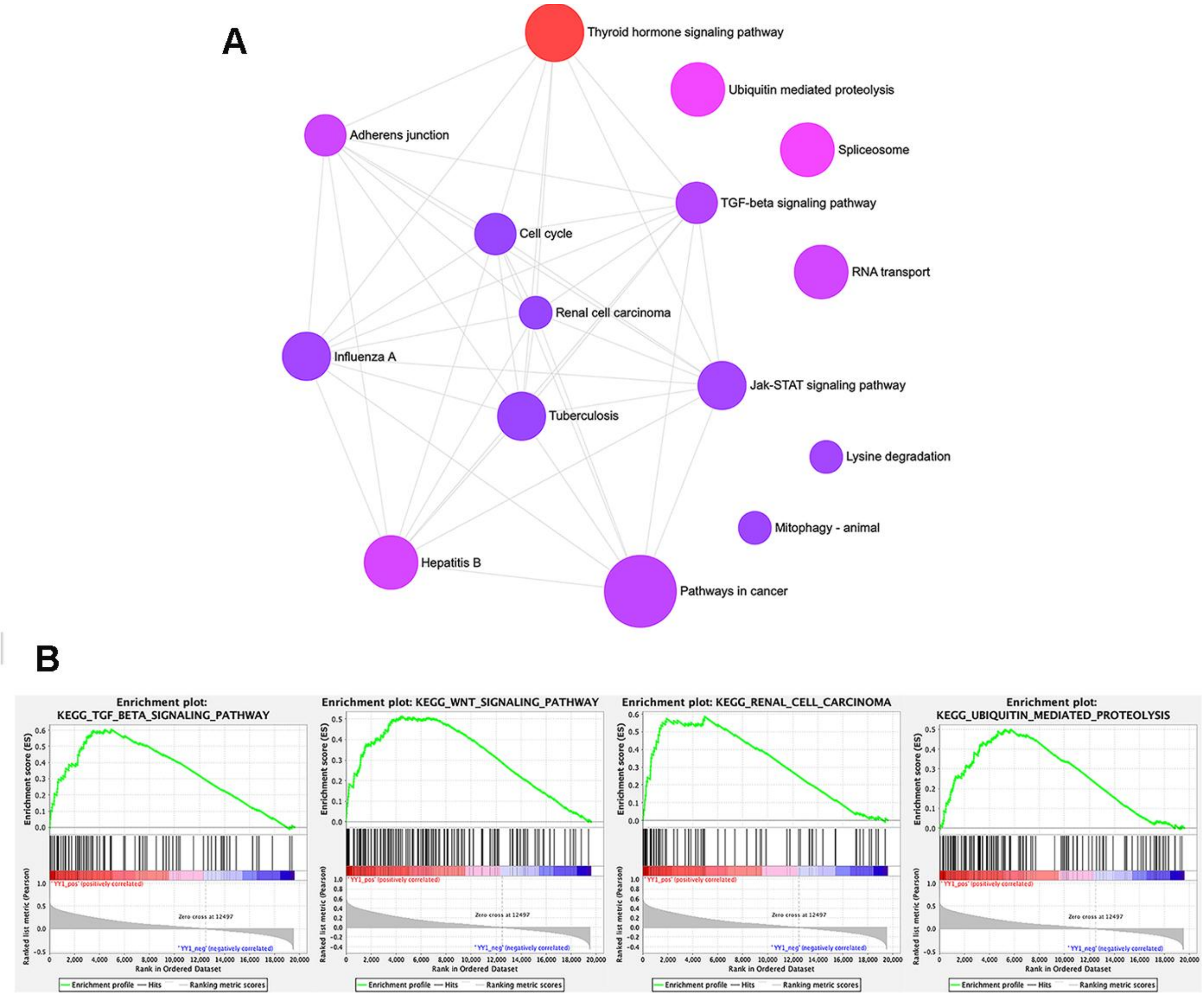

Supplementary Figure 2. GSEA of intersected genes and YY1. (**A**) An interactive network of the top 15 KEGG enriched pathways upon 165 intersected genes. (**B**) Four out of the top 10 KEGG pathway enrichment results of YY1 single gene GSEA.

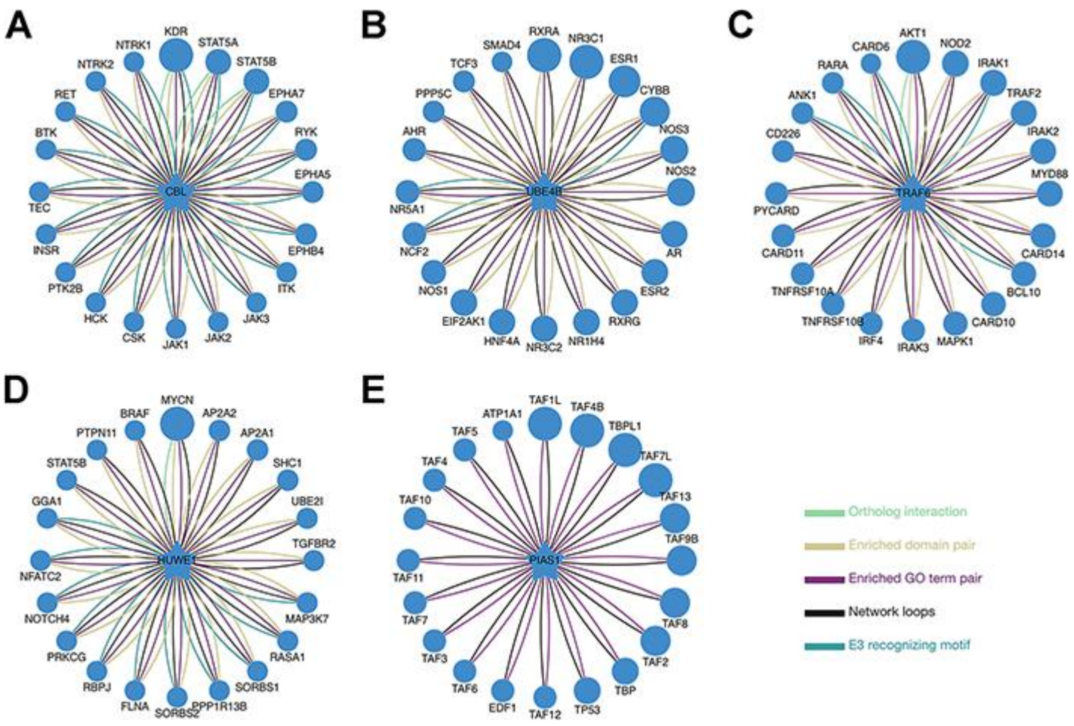

Supplementary Figure 3. Target prediction of E3 ubiquitin ligases. (**A**–**E**) Possible substrates of the five identified E3 ubiquitin ligases predicted by multiple approaches. The size of the target stands for the confidence of their interaction with E3.

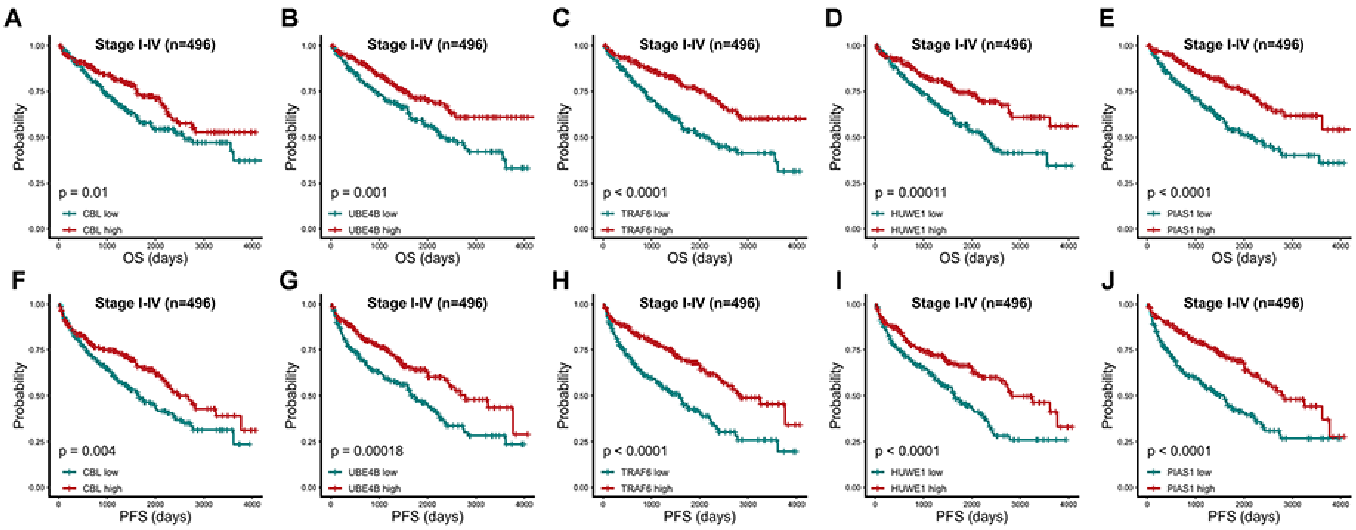

Supplementary Figure 4. Survival analysis of E3 ubiquitin ligases. (**A**–**J**) Overall survival outcomes of 5 E3 ubiquitin ligases upon median cut-off values.

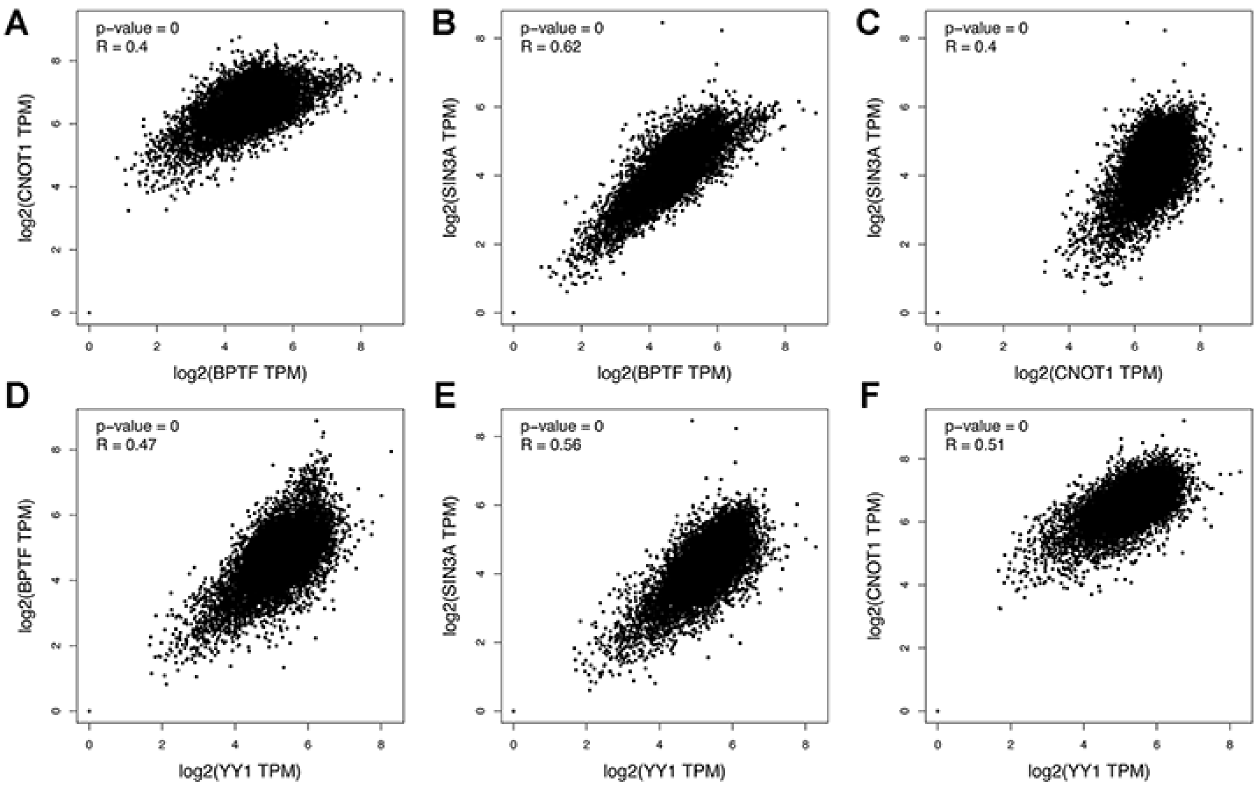

Supplementary Figure 5. TCGA pan-cancer correlation exploration. (**A**–**F**) Pan-cancer Pearson correlation between log2(TPM) expression of BPTF, CNOT1, SIN3A and YY1 performed by GEPIA.

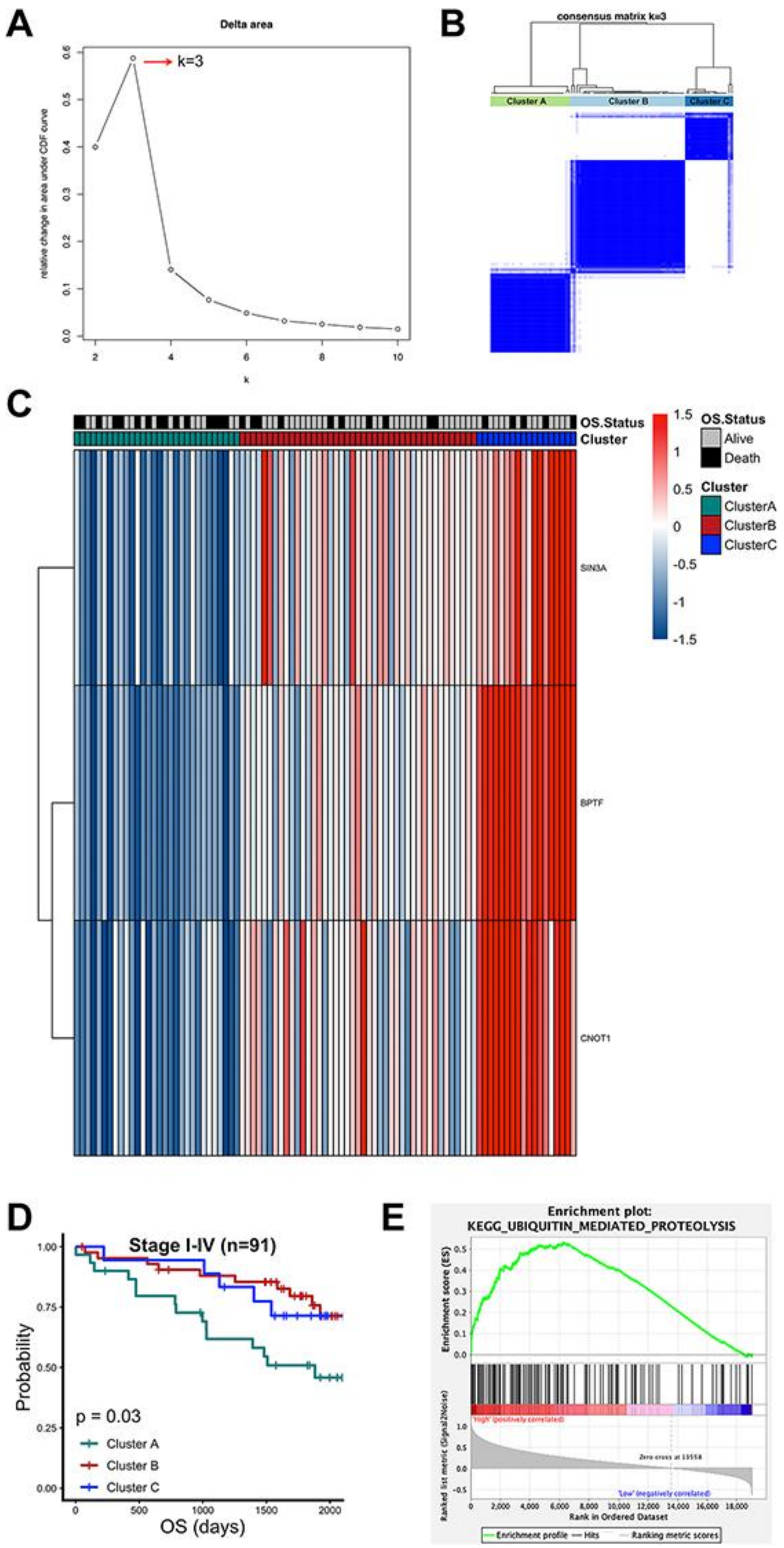

Supplementary Figure 6. ICGC patients grouping based on the expression of BPTF, CNOT1 and SIN3A. (**A**, **B**) Consensus matrix among CDF curves increase from 2 to 10 clusters and groups acquisition. (**C**) The comparison of the expression level of BPTF, CNOT1 and SIN3A among groups. (**D**) Comparison of overall survival outcome among groups. (**E**) KEGG pathways enrichment of patients in cluster B and cluster C.

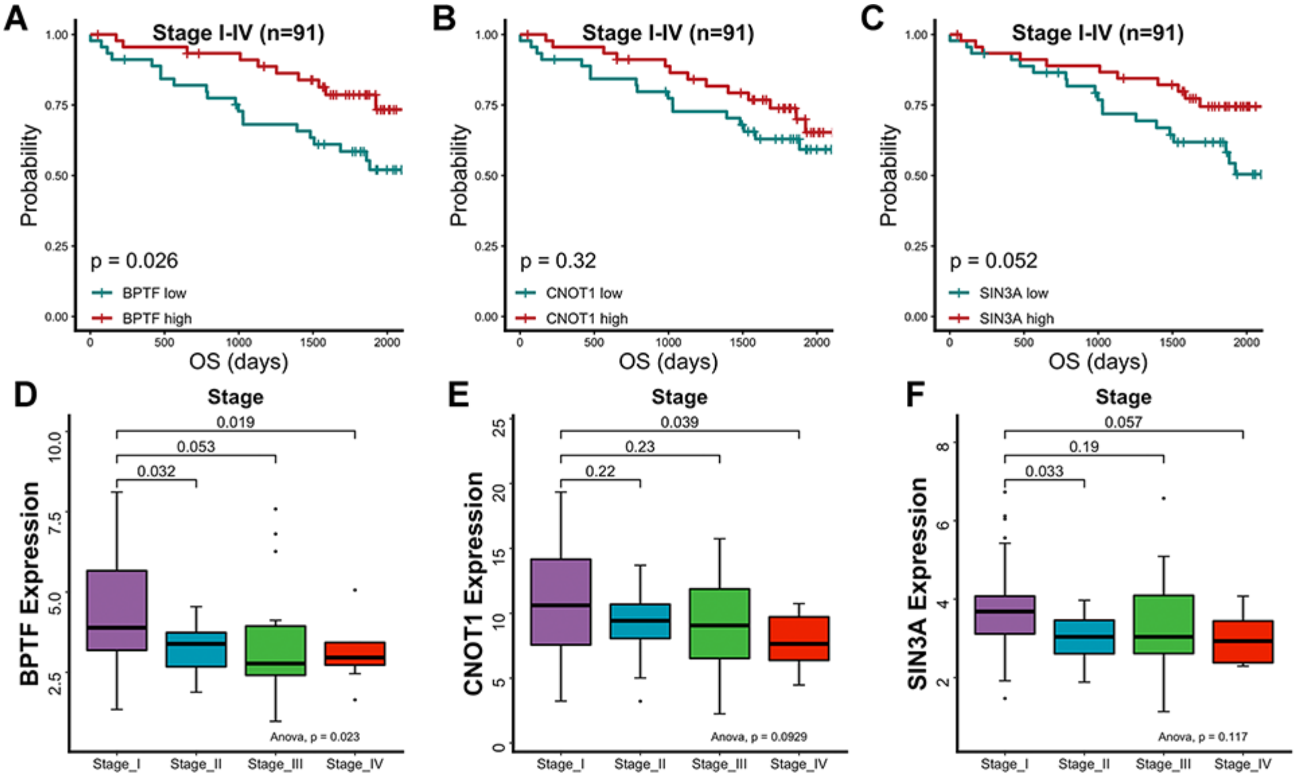

Supplementary Figure 7. Validation of the prognostic values and clinicopathological parameters comparison of BPTF, CNOT1 and SIN3A in ICGC dataset. (**A**–**C**) Validation of the overall survival. (**D**–**F**) Expression level among different stage.

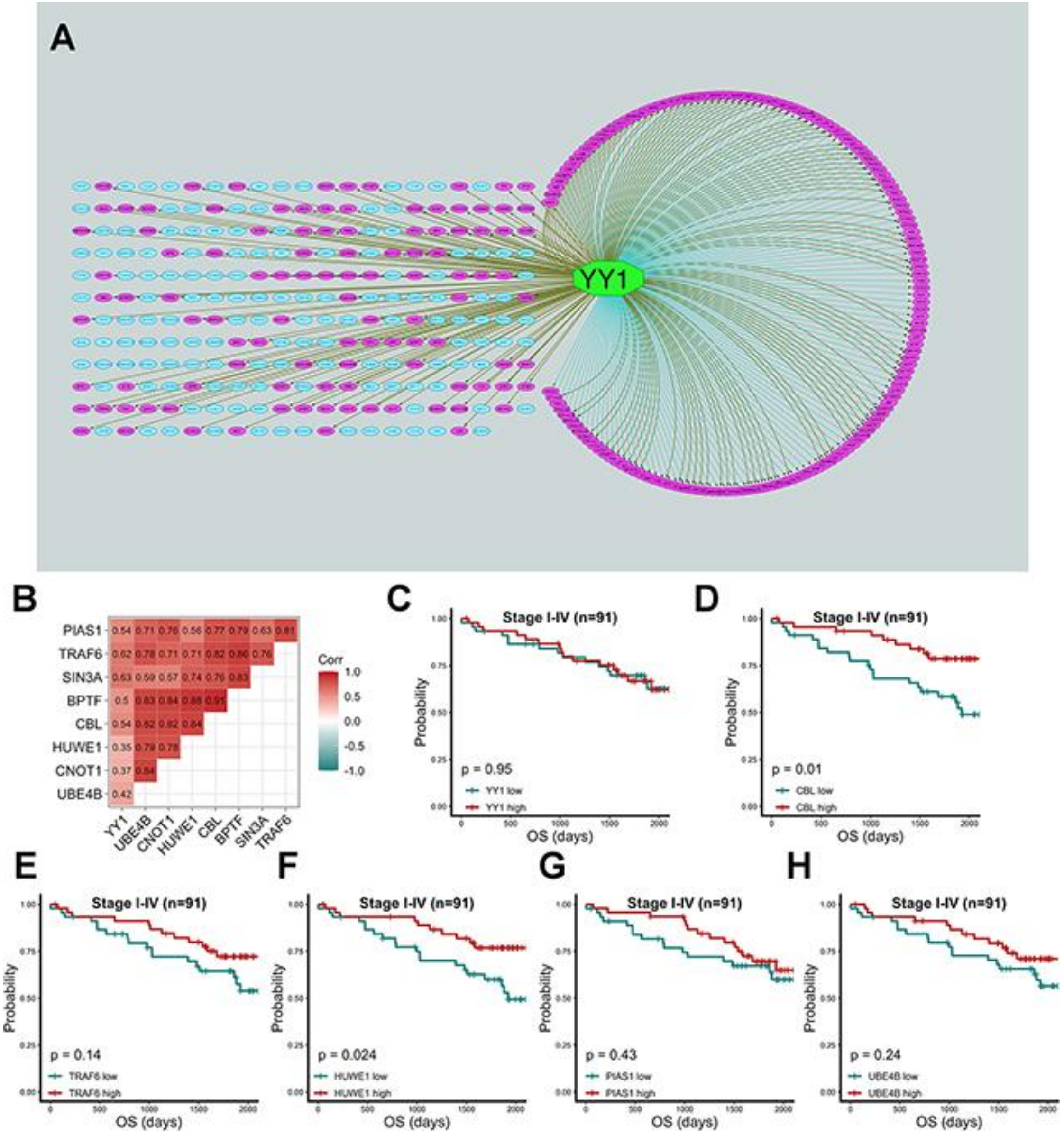

Supplementary Figure 8. Validation of transcriptional factor prediction of correlated genes and prognostic value of E3 ubiquitin ligases in ICGC dataset. (**A**) Transcriptional factor prediction of 398 overlapped correlated genes (Pearson r>0.6, p<0.001) of BPTF, CNOT1 and SIN3A. (**B**) Pearson correlation analysis between BPTF, CNOT1, SIN3A, YY1 and 5 E3 ubiquitin ligases. (**C**–**H**) Overall survival analysis of YY1, CBL, TRAF6, HUWE1, PIAS1 and UBE4B.
