# Supplementary material for: Identification of prognostic chromatin-remodeling genes in clear cell renal cell carcinoma
Source: Aging (Albany NY). 2020 Nov 20;12(24):25614–42. doi: 10.18632/aging.104170 (PMC7803503; doi:10.18632/aging.104170)
Supplement: Supplementary Tables [file aging-12-104170-s002.pdf]

SUPPLEMENTARY TABLES

Supplementary Table 1. Association between Cluster A, Cluster B, clinicopathological parameters and common renal carcinoma genomic mutation characteristics in TCGA-KIRC (n=496).

	Cluster A (n=206)	Cluster B (n=290)	Total (n=496)	P value
**Age**				
**Mean±SD**	61.4 (11.7)	59.8 (12.3)	60.5 (12.1)	0.175
**Gender**				
**Female**	67 (32.5%)	106 (36.6%)	173 (34.9%)	
**Male**	139 (67.5%)	184 (63.4%)	323 (65.1%)	0.406
**Stage**				
**I**	84 (40.8%)	166 (57.2%)	250 (50.4%)	
**II**	22 (10.7%)	30 (10.3%)	52 (10.5%)	
**III**	60 (29.1%)	58 (20.0%)	118 (23.8%)	
**IV**	40 (19.4%)	36 (12.4%)	76 (15.3%)	0.002*
**Grade**				
**I-II**	72 (35.0%)	149 (51.4%)	221 (44.6%)	
**III-IV**	131 (63.6%)	136 (46.9%)	267 (53.8%)	3e-04*
**VHL status**				
**Wild-type**	62 (35.0%)	95 (51.4%)	157 (44.6%)	
**Mutant**	56 (63.6%)	98 (46.9%)	154 (53.8%)	0.652
**PBRM1 status**				
**Wild-type**	69 (33.5%)	108 (37.2%)	177 (35.7%)	
**Mutant**	49 (23.8%)	85 (29.3%)	134 (27.0%)	0.751
**TP53 status**				
**Wild-type**	113 (54.9%)	191 (65.9%)	304 (61.3%)	
**Mutant**	5 (2.4%)	2 (0.7%)	7 (1.4%)	0.146
**MTOR status**				
**Wild-type**	107 (51.9%)	184 (63.4%)	291 (58.7%)	
**Mutant**	11 (5.3%)	9 (3.1%)	20 (4.0%)	0.165

Supplementary Table 2. Referenced model accuracy corresponding to the number of variables included in features selection.

Variables	Accuracy	Kappa	AccuracySD	KappaSD
**2**	0.885	0.76685814	0.15621254	0.31530365
**3**	0.90183333	0.79766067	0.14147878	0.28676197
**4**	0.91633333	0.8291625	0.11966986	0.23714195
**5**	0.91233333	0.81825341	0.12669989	0.26149403
**6**	0.927	0.85192641	0.11011625	0.22121793
**7**	0.95016667	0.89943057	0.09585972	0.19280668
**8**	0.956	0.91039794	0.09246348	0.1873606
**9**	0.94566667	0.88912754	0.09456677	0.19169125
**10**	0.95366667	0.9039627	0.08562768	0.17805629
**11**	0.9695	0.93625874	0.07313161	0.15326379
**12**	0.9695	0.93695804	0.07313161	0.15161329
**13**	0.9655	0.92932401	0.08186432	0.16845566
**14**	0.967	0.93201632	0.08140806	0.16819983
**15**	0.969	0.93516317	0.07970273	0.16676898
**16**	0.9695	0.93631702	0.07846218	0.16410564
**17**	0.9715	0.94086247	0.07662548	0.15939759
**18**	0.9735	0.94465035	0.06906848	0.14463067
**19**	0.9675	0.93317016	0.08020649	0.16558167
**20**	0.9735	0.94395105	0.06906848	0.14639705
**21**	0.9715	0.93946387	0.07662548	0.1625547
**22**	0.9695	0.93631702	0.07846218	0.16410564
**23**	0.9695	0.93631702	0.07846218	0.16410564
**24**	0.9695	0.93631702	0.07846218	0.16410564
**25**	0.96833333	0.93337995	0.07299509	0.15425558
**26**	0.9735	0.94395105	0.06906848	0.14639705
**27**	0.9735	0.94395105	0.06906848	0.14639705
**28**	0.978	0.9527972	0.06289321	0.1354076
**29**	0.9775	0.95164336	0.0645008	0.13876189
**30**	0.9755	0.9477972	0.0668539	0.14268294
**31**	0.978	0.9527972	0.06289321	0.1354076

**32**	0.9755	0.9477972	0.0668539	0.14268294
**33**	0.9735	0.94400932	0.07468959	0.15770879
**34**	0.974	0.94370629	0.06759953	0.14682918
**35**	0.978	0.9527972	0.06289321	0.1354076
**36**	0.96783333	0.93228438	0.07957008	0.16766191
**37**	0.9715	0.93940559	0.07115746	0.15160319
**38**	0.97183333	0.94061772	0.07042022	0.14888174
**39**	0.9755	0.9477972	0.0668539	0.14268294
**40**	0.974	0.94446387	0.07333333	0.15647684
**41**	0.978	0.9527972	0.06289321	0.1354076
**42**	0.9755	0.94785548	0.07264658	0.1542658
**43**	0.974	0.94440559	0.06759953	0.14506925
**44**	0.9755	0.9477972	0.0668539	0.14268294
**45**	0.976	0.94895105	0.06531973	0.13945516
**46**	0.978	0.9534965	0.06289321	0.13344907
**47**	0.976	0.94895105	0.06531973	0.13945516
**48**	0.974	0.94370629	0.06759953	0.14682918
**49**	0.976	0.94900932	0.07123726	0.1512849
**50**	0.976	0.94825175	0.06531973	0.14130776
**51**	0.972	0.93991841	0.07531751	0.16137269
**52**	0.9715	0.93870629	0.07115746	0.15326508
**53**	0.9735	0.94325175	0.06906848	0.14813903
**54**	0.9715	0.93940559	0.07115746	0.15160319
**55**	0.9675	0.93037296	0.08020649	0.1714723
**56**	0.9735	0.94325175	0.06906848	0.14813903
**57**	0.976	0.94825175	0.06531973	0.14130776
**58**	0.972	0.93916084	0.06974702	0.15201314
**59**	0.978	0.9527972	0.06289321	0.1354076
**60**	0.9715	0.93800699	0.07115746	0.15490595
**61**	0.9695	0.93416084	0.07313161	0.15809319
**62**	0.9735	0.94400932	0.07468959	0.15770879
**63**	0.976	0.94900932	0.07123726	0.1512849
**64**	0.978	0.9527972	0.06289321	0.1354076
**65**	0.9775	0.95234266	0.0645008	0.13685734
**66**	0.974	0.94370629	0.06759953	0.14682918
**67**	0.976	0.94895105	0.06531973	0.13945516
**68**	0.976	0.94825175	0.06531973	0.14130776
**69**	0.9675	0.92961538	0.075	0.16264989
**70**	0.976	0.94755245	0.06531973	0.14313293
**71**	0.98	0.95664336	0.06030227	0.13112135
**72**	0.9715	0.93870629	0.07115746	0.15326508
**73**	0.976	0.94825175	0.06531973	0.14130776
**74**	0.978	0.95285548	0.0690191	0.14756065
**75**	0.98	0.95664336	0.06030227	0.13112135
**76**	0.976	0.94755245	0.06531973	0.14313293
**77**	0.974	0.94376457	0.07333333	0.1581101
**78**	0.976	0.94825175	0.06531973	0.14130776
**79**	0.974	0.94446387	0.07333333	0.15647684
**80**	0.9715	0.93870629	0.07115746	0.15326508
**81**	0.98	0.95734266	0.06030227	0.12907677
**82**	0.978	0.9520979	0.06289321	0.1373346
**83**	0.98	0.95664336	0.06030227	0.13112135
**84**	0.9755	0.9470979	0.0668539	0.14448852
**85**	0.9755	0.9477972	0.0668539	0.14268294
**86**	0.9675	0.93037296	0.08020649	0.1714723
**87**	0.976	0.94825175	0.06531973	0.14130776

Supplementary Table 3. Order of the 38 variables to be included according to the highest accuracy of the model.

Variable order	Selected genes
**1**	CNOT1
**2**	SIN3A
**3**	BPTF
**4**	CHD8
**5**	CHD9
**6**	DEK
**7**	SIRT6
**8**	SAP30L
**9**	C6orf89
**10**	SUPT16H
**11**	SMARCA5
**12**	CHD6
**13**	RBBP5
**14**	SMARCA2
**15**	CNOT6L
**16**	CNOT8
**17**	CNOT6
**18**	BAZ1B
**19**	CHD4
**20**	BRMS1
**21**	ACTR8
**22**	INO80B
**23**	BRD3
**24**	CNOT7
**25**	PHF8
**26**	CBX5
**27**	RUVBL2
**28**	TOP1
**29**	ACTR6
**30**	MORF4L1
**31**	CHD1
**32**	ANP32E
**33**	INO80E
**34**	ACTB
**35**	BRD7
**36**	PABPC1L
**37**	SMARCD3
**38**	UTY
**39**	PIH1D1
**40**	DPF2
**41**	BRD1
**42**	CAMK2D
**43**	MIER2

**44**	ACTR5
**45**	PHF1
**46**	SS18
**47**	CNOT3
**48**	DAXX
**49**	BRD8
**50**	SAP30BP
**51**	CHD3
**52**	WDR77
**53**	SUPT20H
**54**	BAZ1A
**55**	WDR61
**56**	NUDT5
**57**	RBBP7
**58**	SAP18
**59**	LRRK2
**60**	INO80C
**61**	ACTN4
**62**	ANP32B
**63**	SIRT2
**64**	CNOT9
**65**	SMARCB1
**66**	HIST1H1C
**67**	PHF21A
**68**	RBBP8
**69**	SMYD2
**70**	SS18L1
**71**	SUPT5H

Supplementary Table 4. TCGA pan-cancer survival analysis of BPTF, CNOT1 and SIN3A.

		**BLCA**	**BRCA**	**CESC**	**CHOL**	**COAD**	**ESCA**	**GBM**
**BPTF**	HR (high)	0.86	1.1	1.2	0.38	0.96	1.2	0.78
	Logrank p	0.32	0.51	0.42	0.049	0.86	0.54	0.17
**SIN3A**	HR (high)	1	1	0.85	0.58	1.2	0.86	0.99
	Logrank p	0.9	0.98	0.48	0.27	0.55	0.51	0.95
**CNOT1**	HR (high)	1	1.1	0.86	0.8	0.67	1.7	0.92
	Logrank p	0.86	0.42	0.53	0.64	0.11	0.03*	0.65

		**HNSC**	**KIHC**	**KIRP**	**LAML**	**LIHC**	**LUAD**	**LUSC**
**BPTF**	HR (high)	0.93	3.8	0.92	1.1	1.4	1.4	1.1
	Logrank p	0.62	0.075	0.78	0.84	0.065	0.025*	0.36
**SIN3A**	HR (high)	1	3.6	1.4	1.2	1.1	1.1	1.1
	Logrank p	0.93	0.087	0.32	0.5	0.6	0.67	0.71
**CNOT1**	HR (high)	1.1	0.45	0.85	1.4	1.3	1.3	1.1
	Logrank p	0.7	0.24	0.6	0.19	0.17	0.12	0.47

		**OV**	**PAAD**	**PRAD**	**READ**	**STAD**	**THCA**	**UCEC**
**BPTF**	HR (high)	1.1	0.76	1.3	0.51	1.1	2.3	1.1
	Logrank p	0.59	0.19	0.72	0.17	0.7	0.12	0.84
**SIN3A**	HR (high)	0.94	1.3	0.79	0.6	1.2	1.7	1.2
	Logrank p	0.59	0.26	0.72	0.28	0.33	0.3	0.59
**CNOT1**	HR (high)	0.95	1.1	1.8	0.53	1.3	1.8	0.98
	Logrank p	0.67	0.53	0.35	0.19	0.083	0.26	0.96
